# Validation of the Spanish version of the Amsterdam Preoperative Anxiety and Information Scale (APAIS)

**DOI:** 10.1186/s12955-017-0695-8

**Published:** 2017-06-07

**Authors:** Manuel Vergara-Romero, José Miguel Morales-Asencio, Angelines Morales-Fernández, Jose Carlos Canca-Sanchez, Francisco Rivas-Ruiz, Jose Antonio Reinaldo-Lapuerta

**Affiliations:** 1 Agencia sanitaria costa del sol, Málaga, Marbella Spain; 2 Faculty of Health Sciences. Universlty of Málaga, Málaga, Spain; 3 Hospital Costa del Sol, Autovia A-7 KM, 187 29603 Málaga, Spain

**Keywords:** Preoperative anxiety, Questionnaires, Validation studies

## Abstract

**Background:**

Preoperative anxiety is a frequent and challenging problem with deleterious effects on the development of surgical procedures and postoperative outcomes. To prevent and treat preoperative anxiety effectively, the level of anxiety of patients needs to be assessed through valid and reliable measuring instruments. One such measurement tool is the Amsterdam Preoperative Anxiety and Information Scale (APAIS), of which a Spanish version has not been validated yet.

**Objective:**

To perform a Spanish cultural adaptation and empirical validation of the APAIS for assessing preoperative anxiety in the Spanish population.

**Methods:**

A two-step forward/back translation of the APAIS scale was performed to ensure a reliable Spanish cultural adaptation. The final Spanish version of the APAIS questionnaire was administered to 529 patients between the ages of 18 to 70 undergoing elective surgery at hospitals of the *Agencia Sanitaria Costa del Sol* (Spain). Cronbach’s alpha, homogeneity index, intra-class correlation coefficient, and confirmatory factor analysis were calculated to assess internal consistency and criteria and construct validity.

**Results:**

Confirmatory factor analysis showed that a one-factor model was better fitted than a two-factor model, with good fitting patterns (root mean square error of approximation: 0.05, normed-fit index: 0.99, goodness-of-fit statistic: 0.99). The questionnaire showed high internal consistency (Cronbach’s alpha: 0.84) and a good correlation with the Goldberg Anxiety Scale (CCI: 0.62 (95% CI: 0.55 to 0.68).

**Conclusions:**

The Spanish version of the APAIS is a valid and reliable preoperative anxiety measurement tool and shows psychometric properties similar to those obtained by similar previous studies.

## Background

Patients undergoing elective surgery often feel anxiety due to the risks involved and the unfamiliarity of the situation. It is estimated that 11% to 92% of patients undergoing surgery experience anxiety [1, 2].

The level of preoperative anxiety felt by each patient varies and is dependent on multiple factors [3]. These include the prognosis of the intervention, fears about the anesthetic and postoperative pain, and concerns about the outcome of the procedure [4, 5]. However, a range of factors related to the sociodemographic and psychosocial characteristics of the patient also need to be considered, for example, personal anxiety levels, personality traits, sensitivity to pain, and the use of coping strategies. Additionally, other important variables include preexisting diseases, the complexity of the intervention, previous surgical experiences, and prior information received [6].

Preoperative anxiety is associated with a negative emotional state and generates a physiological activation of the body to face a perceived risk. This may negatively affect the development of the surgical intervention [3]. It is amply demonstrated that anxiety levels increase before an intervention, triggering a stress response which includes the release of catecholamines, sympathetic hyperactivity, hyper-metabolism, neuroendocrine changes, electrolyte alterations and immunological modifications [7]. Patients with a high level of preoperative anxiety require higher doses of anesthetics and need more peri- and postoperative analgesia [4, 8]. This often results in a longer hospital stay, increases the risk of readmission after surgery and raises morbidity and mortality [9] rates.

These complications make evident that preoperative anxiety needs to be assessed and addressed, supporting the routine administration of preoperative anxiolytics to all surgical patients [10]. Most scales for assessing anxiety (Goldberg Anxiety Scale, GADS [11]; State-Trait Anxiety Inventory, STAI [12]; Depression, Anxiety and Stress Test, DASS [13]; Hospital Anxiety and Depression, HADS [14]; Visual Analogue Scale for Anxiety, VAS-A [15]) have not been validated for surgical patients and do not evaluate their information needs, as they have not been specifically developed to measure preoperative anxiety. Extensive evidence has shown that preoperative information plays a vital role in reducing preoperative anxiety [16]. Interventions such as nursing preoperative visits or psychoeducational and audiovisual interventions that provide patients with additional information on the procedure to take place have shown reduction of anxiety [17–19].

In 1996, Moermann et al. developed the Amsterdam Preoperative Anxiety and Information Scale (APAIS) [20], a self-reported questionnaire specifically validated for assessing preoperative anxiety. APAIS is a useful, easy-to-use, clinically relevant instrument with good acceptance among patients and a simple format that facilitates analysis. This scale has been validated and translated into several languages [21], namely Dutch [20] English [22] German [6] French [23], Japanese [24] Slovak [25], Malay [21], and Indonesian [26]. Although APAIS has been previously used in Spanish studies [27–30], a validated Spanish version did not exist prior to our study.

## Methods

### Aim

The aim of this study was to carry out a Spanish cultural adaptation of the and empirical psychometric validation of the APAIS for the Spanish population.

### Design

A psychometric validation study was conducted for adaptation of the APAIS scale.

### Setting

The study was carried out at hospitals under the management of the *Agencia Sanitaria Costa del Sol* (Spain), a public institution with 350 beds that provide health care to 396.000 habitants. Patients were consecutively recruited between July 2015 and March 2016.

### Study subjects

The sample included patients with ages ranging from 18 to 70 undergoing elective surgery in the specialties of general surgery, urology, gynecology, orthopedics, otolaryngology, ophthalmology and dermatology*.* The study subjects spoke and understood Spanish and voluntarily provided consent to participate in the study. Exclusion criteria were: psychiatric disease and/or cognitive impairment confirmed in clinical records, self-reported severe sensory disability preventing the subject from understanding or undergoing the tests (i.e. severe deafness), or who were not sufficiently proficient in Spanish.

The types of anesthesia administered included general, regional and local anesthesia in a hospitalization regimen of inpatient surgery and major and minor ambulatory surgery.

Some patients could have been administered an anxiolytic drug immediately before surgery, and were asked to complete the APAIS and GADS questionnaires prior to entering the operating room.

Sample size was calculated following the method of MacCallum et al. [31], namely: assuming a null hypothesis of a root mean square error of approximation between 0.04 and 0.08 with an alpha value of 0.05 and a statistical power of 0.8 and a maximum of 18 degrees of freedom, a sample size of 500 patients was obtained. This size was increased by 5% in anticipation of possible loss to follow-ups.

### Description of the measuring instrument and variables

APAIS is a self-reported six-item questionnaire that has been validated for assessing preoperative anxiety. The scale is divided into two subscales exploring three aspects of preoperative anxiety: anesthesia, surgery (items 1, 2, 4 and 5) and need for information (items 3 and 6). Each question is rated on a five-point Likert’s scale, where a value of 1 indicates “not anxious at” all and 5 means “extremely anxious”. The cut-off points for the overall score established by the authors of the original version are 11 and up to 13, when used for research [20]. APAIS has also been reported to be useful as a predictor of early postoperative pain [32].

Patient sociodemographic variables, comorbidities, anesthetic risk (ASA) and history of previous surgery, amongst other data, were recorded. Anxiety was measured using the Spanish version of the Goldberg Depression and Anxiety scales (GADS) [33]. The scale had good psychometric properties (it has a one-dimensional structure that explained 72% of variance, a Cronbach’s alpha of 0.936, and a cut-off point of 10 obtained a sensitivity of 86.8%, and specificity of 93.4%), and confirmed that it could be reliably used by health professionals not specialized in mental health, such as anesthetists and surgical nurses.

### Data collection

Surgical nurses previously trained in the implementation of the scales informed the patients about the procedure and obtained informed consent from patients who met inclusion criteria. Data were collected through face-to-face interviews in the surgical area immediately prior to surgery.

### Ethical issues

This study was approved by the Local Research Ethics Committee Costa del Sol (Spain), CEI (002-ma-PR-APAIS) on 26 March 2015, and was carried out in accordance with the ethical principles as set out in the Declaration of Helsinki. Written consent was obtained from all the participants in the study.

### Spanish cultural adaptation

**Fig. 1:**
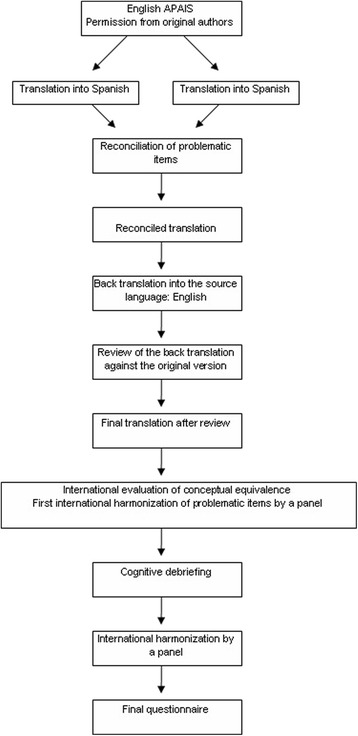
Process of cross-cultural adaptation

It was performed according to the methodology described by Guillemin et al. [34] and ISPOR guidelines [35]. Such method includes the following stages: i) translation of the source version into the target language; ii) use of qualitative methods to check cultural adaptation to the local population; and iii) back translation from the target language to the source language to verify that the underlying meaning of questions was appropriately transferred (Fig.1).

First of all, authorization was obtained from the authors of the original scale to carry out the Spanish cultural adaptation of their scale. Next, two native Spanish language translators prepared draft translations of the original scale separately. The translators reviewed their two translations with the research group and produced an adapted version of the scale. Following this, a blinded back translation was performed on the adapted version and compared with the original scale to resolve discordances. A second adapted version of the questionnaire was then produced. The understandability, clarity and familiarity of the Spanish version were evaluated through cognitive debriefing using inquiry and reformulation techniques (Table 1).

**Table 1:** Translated items of Amsterdam Preoperative Anxiety and Information Scale (APAIS)

Original items	Spain items
1. I am worried about the anaesthetic. 2. The anaesthetic is on my mind continually. 3. I would like to know as much as possible about the anaesthetic. 4. I am worried about the procedure. 5. The procedure is on my mind continually. 6. I would like to know as much as possible about the procedure	1. Estoy preocupado por la anestesia 2. Pienso en la anestesia continuamente 3. Me gustaría saber lo máximo posible acerca de la anestesia 4. Estoy preocupado por la operación 5. Pienso en la operación continuamente 6. Me gustaría saber lo máximo posible acerca de la operación

### Statistical analysis

Descriptive analysis was performed of the sociodemographic and clinical variables. The normality of the distribution of all variables was evaluated by the Kolmogorov-Smirnov test, and the skewness, kurtosis and histograms of the distributions were all ascertained. Bivariate analyses were carried out using the Student’s t and the chi-square tests, in accordance with the characteristics of the variables analysed, when they were normally distributed. Otherwise, non-parametric tests such as the Wilcoxon test and the Mann-Whitney U test were used. One-way ANOVA was used to determine quantitative and qualitative relationships where appropriate, with measures of central robustness in cases of non-homoscedasticity (determined by Levene’s test), using the Welch and Brown-Forsythe tests. If any of the assumptions necessary for the ANOVA test were not met, the Kruskall-Wallis test was performed.

Item-endorsement was determined by observing ceiling and floor effects. Internal consistency was assessed by Cronbach’s alpha; inter-item correlation and homogeneity index were determined. Construct validity was assessed by exploratory factor analysis using both principal axis factoring with oblique rotation (Oblimin), and principal component extraction with Varimax rotation on a randomized sub-sample of 155 subjects. Sample adequacy and level of inter-item correlation were previously evaluated by the Kaiser-Meyer-Olkin test and Bartlett’s sphericity test. Confirmatory factor analysis was subsequently performed both in the 374 resting subjects and in the whole sample, using the following indices of fit: CMIN/DF, root mean square error of approximation index (RMSEA) and its confidence interval (90% CI), normed fit index (NFI), comparative fit index (CFI) and goodness of fit index (GFI). Multivariate normality was determined by Mardia’s coefficient. Statistical analysis was performed using IBM SPSS version 22 and AMOS 21.

## Results

### Content validity

A panel of six experts (an anesthetist, two PhD nurses and three nurses with a master’s degree) reviewed the initial two translations performed by the two native speakers to produce a final version that was back translated into the source language. The panel verified the conceptual equivalence of this version and decided to replace *“intervención”* with *“operación”* (Items 4, 5 and 6), a much more common term in Spanish. Next, 10 patients aged 45 to 65 years equally distributed by age and sex underwent individual semi-structured cognitive debriefing separately. The purpose of cognitive debriefing was to assess the interpretation of questions by respondents, explore if the terms employed were appropriate, and verify that the items were culturally applicable. All respondents described the items of the questionnaire as clear and understandable. Consequently, it was deemed that no items required modification due to misinterpretation or lack of understanding.

### Empirical validation

**Fig. 2:**
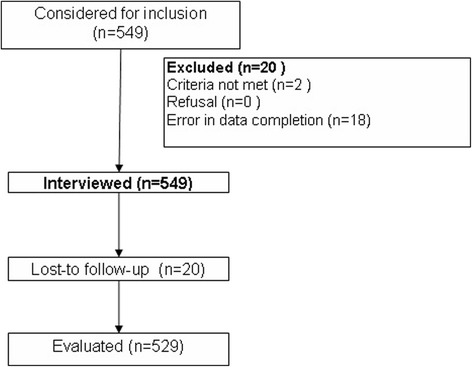
Patient flowchart

Of the 549 subjects recruited, 20 were excluded for not meeting inclusion criteria, and 18 due to errors in questionnaire completion (Fig. 2). The characteristics of the 529 study subjects are detailed in Table 2, being general surgery the highest proportion of patients (*n* = 129; 24.4%), with a mean age of 50.6 years (SD: 14.54), 288 (54.4%) were men, of whom 54 (18.8%) used anxiolytics regularly, versus 5.8% in women (*p* < 0.0001).

**Table 2:** Characteristics of the sample

	Male 288 (54.4%)	Female 241 (41.6%)	Total (*n* = 529)	
	*Mean (SD) or n (%)*	*Mean (SD) or n (%)*	*Mean (SD) or n (%)*	*p*
Age	50.31(13.87)	50.95(15.326)	**50.60(14.54)**	0.902
Regular anxiolytic treatment	54(18.8)	14(5.8)	**68(12.9)**	*<0.0001*
Education level				0.070
No education	37(12.8)	32(13.3)	**69(13.0)**	
Primary Education	113(39.2)	98(40.7)	**211(39.9)**	
Secondary Education	110(38.2)	72(29.9)	**182(34.4)**	
Higher Education	28(9.7)	39(16.2)	**67(12.7)**	
Specialty				*<0.0001*
Surgery	69(24)	60(24.9)	**129(24.4)**	
Urology	23(8)	60(24.9)	**83(15.7)**	
Gynecology	61(21.2)	-	**61(11.5)**	
Traumatology	54(18.8)	52(21.6)	**106(20.0)**	
ENT	16(5.6)	12(5)	**28(5.3)**	
Ophtalmology	32(11.1)	37(15.4)	**69(13)**	
Dermatology	33(11.5)	20(8.3)	**53(10.0)**	
Procedure				0.750
Surgery	40(13.9)	37(15.4)	**77(14.6)**	
Diagnostic surgery	26(9.0)	18(7.5)	**44(8.3)**	
Non-oncological surgery	222(77.1)	186(77.2)	**408(77.1)**	
Hospitalization regimen				0.174
Inpatients	184(63.9)	171(71.0)	**355(67.1)**	
Ambulatory Major Surgery	55(19.1)	41(17.0)	**96(18.1)**	
Outpatient	49(17.0)	29(12.0)	**78(14.7)**	
History of previous surgery				0.701
Yes	215(74.9)	177(73.4)	**392(74.2)**	
No	72(25.1)	64(26.6)	**136(25.8)**	
Have been informed on diagnosis				0.454
Yes	279(96.9)	236(97.9)	**515(97.4)**	
No	9(3.1)	5(2.1)	**14(2.6)**	
Have been informed on the procedure				0.393
Yes	254(88.2)	217(90.0)	**471(89.0)**	
No	34(11.8)	23(9.5)	**57(10.8)**	
Anesthetic risk				0.002
ASA I	106(36.8)	103(42.7)	**209(39.5)**	
ASA II	161(55.9)	101(41.9)	**262(49.5)**	
ASA III	21(7.3)	36(14.9)	**57(10.8)**	
ASA IV	-	1(0.4)	**1(0.2)**	
Type of Anesthesia				*<0.0001*
General	113(39.2)	66(27.4)	**179(33.8)**	
Loco-regional	81(28.1)	111(46.1)	**192(36.3)**	
Local	94(32.6)	64(26.6)	**158(29.9)**	

According to the scores obtained from GAD7, anxiety levels in our study subjects were generally low (mean: 1.79; SD: 2.63), being higher in men with regards to women (2.35; SD; 2.93 vs 1.12; SD: 2.02; *p* < 0.001). No significant differences were observed in anxiety levels between the group that received anxiolytics prior to the procedure and the group who did not receive any drug (1.53; SD: 2.45 vs 1.58; SD: 2.70; *p* = 0.07). The highest anxiety levels were observed in patients undergoing gynecological surgery (2.72; SD: 3.18; *p* < 0.0001), receiving general anesthetics (2.17; SD: 2.78; *p* = 0.001), or using anxiolytics regularly (mostly men) (2.96 SD: 3.11 vs 1.62; SD: 2.51 *p* = 0.001) [36, 37]. Conversely, no significant differences in anxiety levels were observed regarding age, education level or history of previous surgery. No differences were found either between anxiety level and anesthetic risk (ASA I; 2.05; SD: 2.88; ASA II: 1.67; SD: 2.46; ASA III: 1.39; SD: 2.41; *p* = 0.121).

### Characteristics of the items and internal consistency of the APAIS instrument

No ceiling or floor effects were detected in any item. No cases of score bunching above 85% were detected. The mean score on the scale was 12.87 (SD: 6.08) (range of possible values: 5–30). All items showed item-to-total correlations >0.20 (mean: 0.49) (Table 3). Cronbach’s alpha was 0.84 (Table 4).

**Table 3:** Inter-item correlation matrix

Item	1	2	3	4	5	6
1. I am worried about the anesthetic	1000					
2. The anaesthetic is on my mind continually	0.822	1.000				
3. I would like to know as much as possible about the anaesthetic	0.451	0.434	1000			
4. I am worried about the procedure	0.513	0.461	0.325	1000		
5. The procedure is on my mind continually	0.517	0.556	0.351	0.791	1.000	
6. I would like to know as much as possible about the procedure	0.270	0.260	0.580	0.527	0.511	1.000

**Table 4:** Item-total correlation

	Corrected Item-to-total correlation	Squared multiple correlation	Cronbach’s alpha if item removed
I am worried about the anaesthetic	0.650	0.713	0.821
The anesthetic is on my mind continually	0.648	0.713	0.823
I would like to know as much as possible about the anaesthetic	0.550	0.455	0.840
I am worried about the procedure	0.693	0.674	0.812
The procedure is on my mind continually	0.723	0.687	0.806
I would like to know as much as possible about the procedure	0.558	0.507	0.841

### Construct validity

**Fig. 3:**
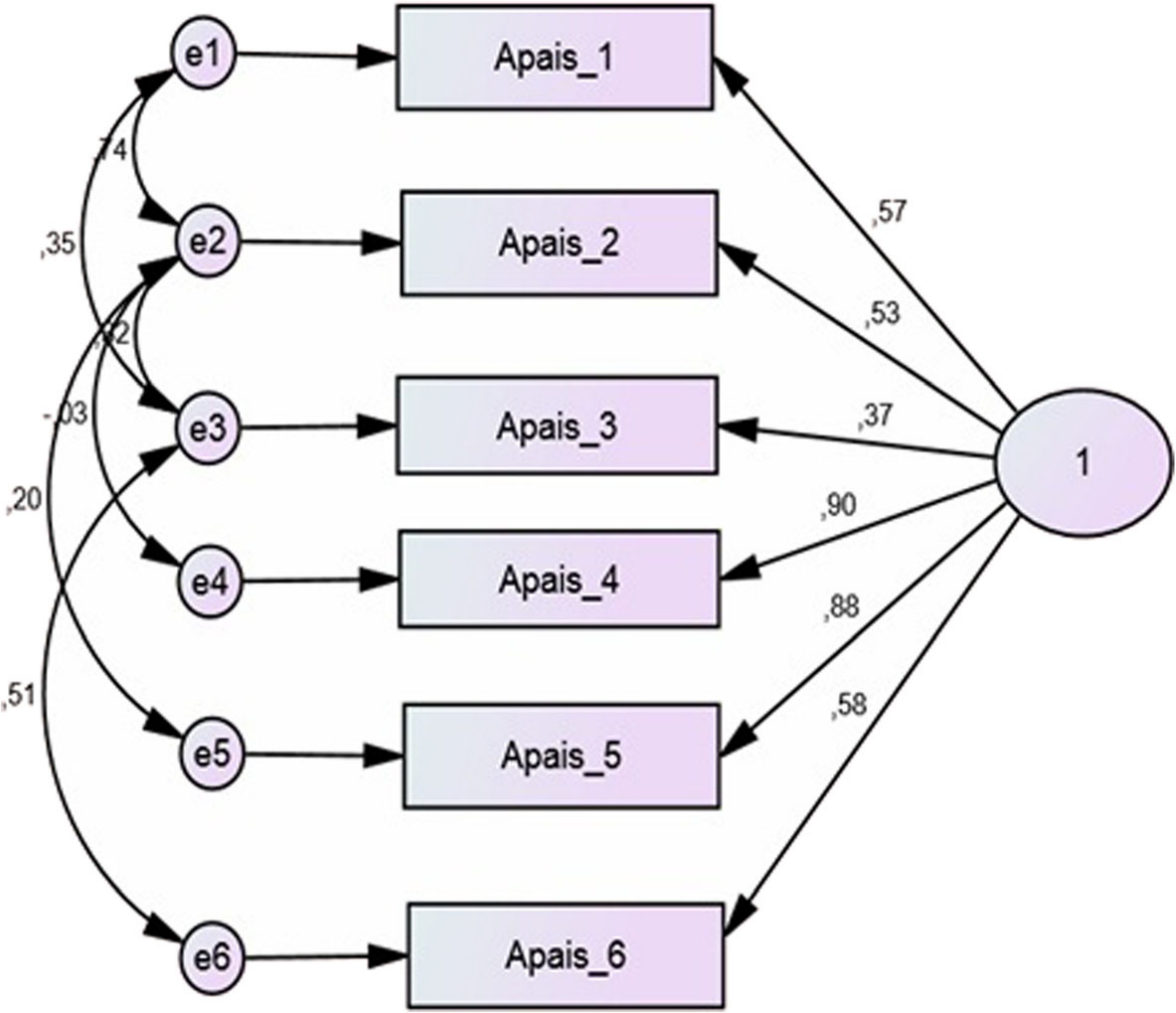
Instrument structure

Exploratory factor analysis (EFA) by oblique rotation (Oblimin) with 155 subjects, yielded a two-factor model that explained 75.78% of variance, whereas exploratory factor analysis of main axis with Varimax rotation produced a two-factor model that explained 74.8% (75%) of variance. Confirmatory factor analysis (CFA) showed that the one-factor model was better fitted than the two-factor model based on EFA, with good fitting patterns (Fig. 3). Multinormality analysis confirmed the validity of parameters (Table 5).

**Table 5:** Confirmatory factor analysis adjustment parameters

	Original	Two-factor model	One-factor model
CMIN/DF	28.29	22.49	2.40
GFI	0.88	0.92	0.99
NFI	0.87	0.92	0.99
CFI	0.88	0.93	0.99
RMSEA (90%CI)	0.48 (0.46 to 0.50)	0.202 (0.17 to 0.23)	0.05 (0.001 to 0.11)

### Criteria validity

**Fig. 4:**
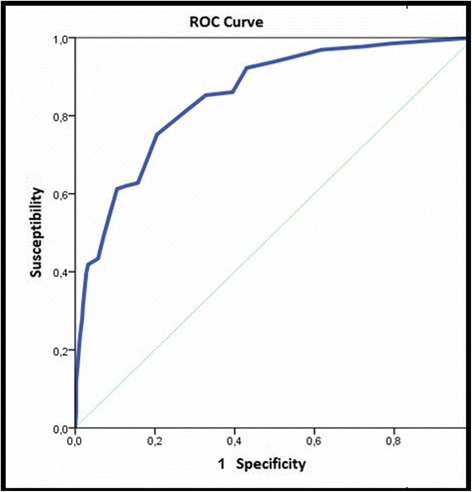
Predictive validity using goldberg as gold standard

Criteria validity was tested by means of ICC and ROC area from APAIS scores and Goldberg Depression and Anxiety scale, obtaining an intra-class correlation coefficient (ICC) of 0.62 (95% CI: 0.55 to 0.68). The area under the curve (Fig. 4) for anxiety, as assessed by the APAIS scale, was 0.85 (95% CI: 0.81 to 0.88) with a cut-off point of 14.

## Discussion

The objective of this study was to carry out a Spanish cultural adaptation and validation of the Spanish version of the APAIS scale. The methods used were consistent with the guidelines established in the literature [34, 35]. An equivalent Spanish version in conceptual and semantic terms was produced. The validation process revealed the strong psychometric properties of the Spanish version concerning reliability and validity.

The internal validity of the scale was tested using exploratory factor analysis (EFA). Estimations were initially performed for a two-factor model, as in the original version. However, confirmatory factor analysis showed that the two-factor model from EFA did not fit the data well. This is frequent when robust factor analysis methods are used, since they usually show fits that are otherwise unacceptable with exploratory methods [38]. Therefore, a one-factor model was used.

The validity and reliability of the Spanish version is similar to that reported by the authors of the original instrument [20]. It is worth mentioning that anxiety and information needs were integrated into a single factor in the Spanish version, as they were considered different manifestations of the same latent variable. This modification may be related to cultural aspects, such as cultural beliefs about fear, or ways of coping to threats, or general knowledge about the surgical and anesthetic process, which evidences that the Spanish version of the scale required a cultural adaptation [39].

The anxiety levels detected in our study were low despite the fact that interviews were conducted immediately before surgery, which has been reported as the point of maximum anxiety in previous studies [7]. This could explain that in the criterion validity process, we found the best sensitivity and specificity values with a cut-off point slightly greater than the original authors [20].

Although no significant differences were observed between the group that received anxiolytics and the group which did not, the results obtained are consistent with those reported in previous studies. The patients undergoing gynecological surgery, receiving general anesthetics and using anxiolytics regularly (mostly men) were the subjects who showed the highest levels of anxiety. Interestingly, the relationship found between general anesthetics and anxiety was not observed in the French APAIS version [23], in which no correlation was found between the type of anesthetic and the levels of anxiety. On the other hand, the finding that men exhibited higher levels of anxiety might be explained by the fact that men took anxiolytic medication more frequently than women and previous studies have documented higher levels of preoperative anxiety in patients who regularly take psychopharmacological substances [37].

Differences by age, education level, or history of previous surgery were not significant, which is in agreement with the results obtained for the Japanese [24] and German [6] validation studies. These findings are consistent with those reported by other authors. It is important to point out that no differences were found between anxiety level and anesthetic risk. This means that perceived threat is independent from the risk factors that could be present prior to the surgery, so that interventions to prevent anxiety should not be guided by this criterion.

Unlike the original scale and subsequent versions in other languages, the validity of criteria of the Spanish APAIS scale was tested against GADS [11], with good results.

One limitation of this study was that the questionnaires were completed immediately prior to entering the operating room. It is possible that preoperative anxiety levels could variate depending on the moment of the evaluation (the imminence of the intervention could trigger the perception of threat and, thus, the anxiety level). Otherwise, the waiting time before the intervention has been reported as an important factor that influences preoperative anxiety [40], although this factor was not evaluated in our study.

## Conclusion

This study confirms the validity and reliability of the Spanish version of the APAIS, showing it to be a useful, brief, clinically relevant instrument with high levels of acceptance among patients given in a format that facilitates analysis. The properties of this scale make its use possible as a standard measurement instrument for assessing preoperative anxiety, especially if validated versions are used.

## Abbreviations

APAIS: the Amsterdam Preoperative Anxiety and Information Scale
CFI: Comparative fit index
CI: Confidence interval
CMIN/DF: Root mean square error of approximation index (RMSEA) divided by degrees of freedom.
DASS: Depression, anxiety and stress test
*EFA*: Exploratory factor analysis
GADS: Goldberg anxiety scale
GFI: Goodness of fit index (GFI).
HADS: Hospital anxiety and depression
*ICC*: Intraclass correlation coefficient
NFI: Normed fit index
RMSEA: Root mean square error of approximation index
STAI: State-Trait anxiety inventory
VAS-A: Visual analogue scale for anxiety
